# Hyponatremia and anti-diuretic hormone in Legionnaires’ disease

**DOI:** 10.1186/1471-2334-13-585

**Published:** 2013-12-11

**Authors:** Philipp Schuetz, Sebastian Haubitz, Mirjam Christ-Crain, Werner C Albrich, Werner Zimmerli, Beat Mueller

**Affiliations:** 1 Medical University Clinic, Kantonsspital Aarau, Aarau, Switzerland; 2 Department of Internal Medicine, Division of Endocrinology, Diabetes and Clinical Nutrition, University Hospital Basel, Basel, Switzerland; 3 Medical University Clinic, Kantonsspital Liestal, Liestal, Switzerland

**Keywords:** SIADH, *Legionella*, Hyponatremia, Low sodium levels, Community-acquired pneumonia

## Abstract

**Background:**

Medical textbooks often list Legionnaires’ disease as a differential diagnosis of the syndrome of inappropriate secretion of anti-diuretic hormone (ADH) (SIADH), but evidence supporting this association is largely lacking. We tested the hypothesis whether hyponatremia in patients with Legionnaires’ disease would be caused by increased CT-ProVasopressin.

**Methods:**

We measured CT-ProVasopressin and sodium levels in a prospective cohort of 873 pneumonia patients from a previous multicentre study with 27 patients having positive antigen tests for Legionella pneumophila.

**Results:**

Patients with Legionnaires’ disease more frequently had low sodium levels (Na < 130 mmol/L) (44.4% vs 8.2%, p < 0.01), but similar mean CT-ProVasopressin levels (pmol/l) (39.4 [±7] vs 51.2 [±2.7], p = 0.43) as compared to patients with pneumonia of other etiologies. In patients with Legionnaires’ disease, CT-ProVasopressin levels showed a positive correlation with sodium (r = 0.42, p < 0.05). Independent of pneumonia etiology, CT-ProVasopressin correlated significantly with the pneumonia severity index (r = 0.56, p < 0.05), ICU admission (adjusted odds ratio per decile, 95% CI) (1.6, 1.2 - 2.0), and 30-day-mortality (1.8, 1.3 - 2.4).

**Conclusion:**

While Legionnaires’ disease was associated with hyponatremia, no concurrent increase in CT-ProVasopressin levels was found, which argues against elevated ADH levels as the causal pathway to hyponatremia. Rather, Vasopressin precursors were upregulated as response to stress in severe disease, which seems to overrule the osmoregulatory regulation of ADH.

## Background

Low sodium levels are a common feature of patients with community-acquired pneumonia (CAP), particularly if caused by *Legionella pneumophila*[1-4]*.* In a retrospective study comparing clinical features of CAP caused by *Legionella* with CAP of other aetiology, low sodium levels (<131 mmol/L) were present in 46% of patients with Legionnaires’ disease as compared to only 14% in patients with CAP of other aetiology [3]. Yet, the physiopathological mechanisms underlying this sodium imbalance remain unclear and evidence from controlled studies is largely lacking. Previous smaller studies found evidence for dysregulation of anti-diuretic hormone (ADH) causing the syndrome of inappropriate secretion of ADH (SIADH) in patients with tuberculosis. This was evidenced by detectable circulating levels of ADH despite low sodium levels in patients [5,6]. More recent reports linked inflammation to low blood sodium levels through an immuno-neuroendocrine pathway with non-osmotic release of vasopressin by interleukin (IL)-6 and other cytokines (reviewed in [7]). Other studies again found that changes in arterial P_a_CO_2_ and oxygenation stimulated hormone release from the posterior and anterior pituitary gland resulting in sodium disturbances [8]. Finally, a direct renal involvement in patients with Legionnaires’ disease causing renal salt wasting has been postulated [9].

Correctly diagnosing the underlying cause of low sodium levels has important therapeutic consequences. Whereas patients with sepsis clearly benefit from early fluid resuscitation [10], free water restriction is recommended in patients with SIADH, because of the relative excess of free water to solute caused by the antidiuretic hormone. Finally, hyponatremia accounts for considerably increased morbidity and mortality, mainly due to brain-function alterations. Groups at increased risk are pre-menopausal females, children, and patients with liver disease and hypoxia [11-15].

Despite the lack of strong empirical evidence linking low sodium levels to a specific pathophysiological pathway, medical textbooks often list Legionnaires’ disease as a differential diagnosis of SIADH. Diagnosing SIADH is challenging in clinical practice, partly because of the analytical challenges of ADH measurement [16]. With the recent availability of an immuno-assay that measures the more stable ADH precursor peptide CT-ProVasopressin showing a close correlation with ADH blood levels [17,18], we sought to investigate whether elevated ADH precursor levels would causatively explain the typical low blood sodium levels of patients with Legionnaires’ disease in a large and well defined cohort of CAP patients from a previous trial [19]. Particularly, we tested the hypothesis that patients with Legionella CAP and low sodium levels would display increased levels of CT-ProVasopressin.

## Methods

### Setting and population studied

The present study used data from 873 patients from a cohort of 925 patients with radiologically confirmed CAP, who had a sodium level measured on admission and a left over blood sample for later measurement of CT-ProVasopressin levels. A detailed description of the previous study has been published elsewhere [19,20] and was published in the clinicaltrials.gov database (NCT00350987). In brief, this was a multicenter, randomized-controlled non-inferiority trial investigating the effects of using procalcitonin for antibiotic stewardship compared to guideline recommendations. Patients with lower respiratory tract infections admitted to emergency departments of one of six hospitals in Switzerland between December 2006 and March 2008 were consecutively included. The primary endpoint was the combined medical failure rate of patients. A study website provided information on the evidence-based management of all patients based on the most recent guidelines [21-24] and explicitly specified the need for X-ray confirmation of CAP, collection of two sets of pre-treatment blood cultures as well as urinary antigen tests for diagnosis of Legionnaires’ disease.

The ethical committee of Basel (EKBB), Switzerland approved the study and all patients gave written informed consent.

### Participants

Inclusion criteria for patients were written informed consent, age ≥ 18 years and admission from the community or a nursing home with the main diagnosis of CAP. Exclusion criteria were the inability to provide written informed consent, insufficient German language skills, active intravenous drug use, previous hospitalisation within 14 days, severe immunosuppression other than corticosteroids, accompanying chronic infection or endocarditis and severe medical co-morbidity where death was imminent. CAP was defined by the presence of at least one respiratory symptom (cough, with and without sputum production, dyspnea, tachypnea, pleuritic pain) plus one auscultatory finding or one sign of infection (core body temperature > 38.0°C, shivers, white blood count > 10 G/L or < 4 G/L cells) and a new infiltrate on chest radiograph [24,25].

### Definitions

In all CAP patients, the protocol specified to perform a qualitative immunochromatographic antigen test for *Legionella pneumophila* serogroup 1 on urine samples on admission (BinaxNow Legionella; Binax). In patients with high suspicion for Legionnaires’ disease despite negative urinary antigen, additional microbiological tests (culture results from respiratory specimen or PCR from respiratory specimen) were performed based on the discretion of the treating physician. Thus the gold standard for diagnosis of Legionnaires’ disease in this study was the clinical diagnosis of a CAP with an infiltrate on chest X-ray and at least one positive microbiological test for *Legionella* (urinary antigen testing, culture results from respiratory specimen or PCR from respiratory specimen). In addition, the protocol specified to collect two pairs of blood cultures under both aerobic and anaerobic conditions before starting antibiotic therapy. Blood cultures were processed using an automated colorimetric detection system (BacT/ALERT, bioMerieux, Durham, NC, USA) in three hospitals and an equivalent blood culture system (BACTEC Becton-Dickinson, Cockeysville, Md.) in the other three hospitals [26]. If blood culture bottles indicating bacterial growth, samples were Gram stained and sub-cultured. The correct identification of the pathogen was achieved according to routine laboratory procedures.

### Clinical examination and laboratory data

In all patients on admission, a thorough clinical examination was performed and two prognostic scores (Pneumonia Severity Index (PSI) and the CURB-65) were calculated [27,28]. For all patients laboratory results were collected from the routine blood analysis on admission including markers of infection (PCT, CRP and WBC) and plasma sodium concentrations. CT-ProVasopressin (pmol/L) was detected in stored EDTA plasma samples of all patients with a new sandwich immunoassay (B.R.A.H.M.S Sevapressin®LIA, B.R.A.H.M.S AG, Hennigsdorf/Berlin, Germany). Briefly, for the assay 50 μl of either serum or plasma are required and no extraction steps or other pre-analytical procedures are necessary. The analytical detection limit is 0.4 pmol/L and the functional assay sensitivity (< 20% inter assay CV) was < 1 pmol/L. In 200 healthy individuals CT-ProVasopressin plasma concentration had a median of 3.7 pmol/L [29].

### Statistical analysis

Discrete variables are expressed as counts (percentage) and continuous variables as medians and interquartile ranges (IQR) or means and standard deviations (SD). Frequency comparison was done by chi-square test. Two-group comparison Mann–Whitney-U test was used. Receiver-operating-characteristics (ROC) were calculated using STATA 9.2 (Stata Corp, College Station, Tex). All testing was two-tailed and P values less than 0.05 were considered to indicate statistical significance.

## Results

### Baseline parameters

Of a total of 873 CAP patients, 31 (3.6%) had a final diagnosis of Legionnaires’ disease. Of these, 4 patients were excluded due missing CT-ProVasopressin levels leaving 27 patients (3.1% of all CAP patients) for the final analysis, All Legionella patients had a positive urine antigen test. *Legionella pneumophila* was also cultured from respiratory secretions in three cases. There were no patients with Legionnaires’ disease which was detected by other means than urinary antigen test. In the overall CAP cohort, a total of 69 patients (7.9%) had positive blood cultures, mainly with growth of *Streptococcus pneumoniae* (86%), none with *Legionella sp*.

Patients with Legionnaires’ disease were similar in terms of age and comorbidities compared to those with CAP of other etiology (Table 1). The acuity of disease on presentation at the emergency room tended to be higher in Legionella patients for some clinical parameters such as body temperature and markers of inflammation (C-reactive protein). Also, clinical outcomes tended to be worse in patients with Legionnaires’ disease with significantly higher ICU admission rates and a trend for longer length of hospital stays. Yet, no significant difference in severity of illness scores (PSI, CURB65) was detected.

**Table 1:** Baseline characteristics (n = 873)

**Parameter**	**All CAP patients**	**Legionnaires’ disease**	**Other CAP etiology**	***p***
	**n = 873**	**n = 27**	**n = 846**	
**Demographics**				
Age (years), mean (SD)	68.3 (±0.6)	64.9 (±3.1)	68.4 (±0.6)	*0.35*
Male gender, n (%)	512 (58.7%)	20 (74.1%)	492 (58.2%)	*0.09*
**Comorbidities, n (%)**				
Congestive heart failure	151 (17.3%)	2 (7.4%)	149 (17.6%)	*0.16*
COPD	266 (30.5%)	3 (11.1%)	263 (31.1%)	*0.03*
Diabetes	149 (17.1%)	6 (22.2%)	143 (16.9%)	*0.47*
Tumor	109 (12.5%)	2 (7.4%)	107 (12.7%)	*0.42*
Chronic renal failure	195 (22.3%)	7 (25.9%)	188 (22.2%)	*0.65*
**Clinical presentation, mean (SD)**				
Heart rate (beats/min)	95.7 (±0.7)	101.3 (±3.9)	95.5 (±0.7)	*0.12*
Blood pressure systolic (mmHg)	133.1 (±0.8)	136.5 (±4.1)	133 (±0.8)	*0.45*
Respiratory rate (breaths/min)	22.1 (±0.3)	25.7 (±3.4)	22 (±0.3)	*0.052*
Temperature (°C)	38 (±0.1)	38.7 (±0.2)	38 (±0.1)	*0.012*
**Blood analysis on admission, mean (SD)**				
Sodium (mmol/l)	135.3 (±0.2)	131.6 (±0.9)	135.4 (±0.2)	*<0.001*
Sodium < 130 mmol/l, n (%)	81 (9.3%)	12 (44.4%)	69 (8.2%)	*<0.001*
C-reactive protein (mg/l)	178.4 (±4.5)	334.2 (±23.6)	173.4 (±4.4)	*<0.001*
Procalcitonin (µg/l)	4.4 (±0.5)	4.1 (±0.9)	4.4 (±0.5)	*0.92*
CT-ProVasopressin (pmol/l)	50.9 (±2.6)	39.4 (±7)	51.2 (±2.7)	*0.43*
**Risk scores on admission, mean (SD)**				
PSI score	92 (±1.2)	101.4 (±6)	91.7 (±1.2)	*0.17*
CURB65 score	1.5 (±0)	1.4 (±0.2)	1.5 (±0)	*0.61*
**Hospital outcomes**				
Mortality, n (%)	49 (5.6%)	2 (7.4%)	47 (5.6%)	*0.68*
ICU admission, n (%)	79 (9.1%)	9 (33.3%)	70 (8.3%)	*<0.001*
Length of stay (days), mean (SD)	9.8 (±0.3)	12.3 (±1.8)	9.7 (±0.3)	*0.09*

### CT-ProVasopressin and sodium levels

**Figure 1:**
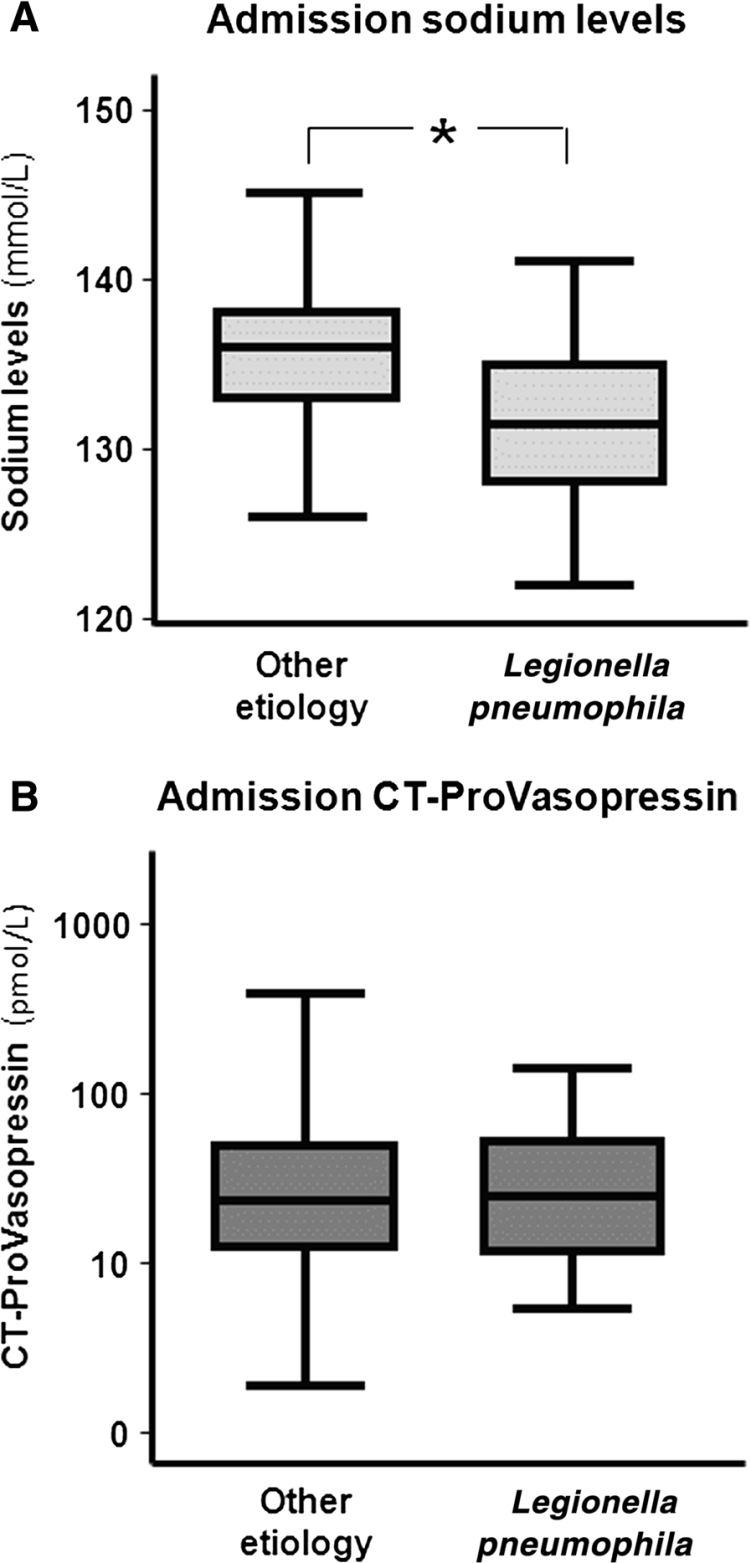
**Admission sodium (A) and CT-ProVasopressin (B) levels in Legionnaires’ disease, and CAP of other (unknown) etiology.**Legend: The lines indicate median values, the boxes indicate upper and lower quartiles of the data, while the whiskers indicate the minimum and maximum values. * p < 0.01.

Mean (SD) sodium levels were significantly lower in patients with Legionnaires’ disease (131.6 mmol/l (±0.9) vs. 135.4 mmol/l (±0.2), p < 0.001) as compared to patients with other CAP etiologies. A total of 44.4% of patients with Legionnaires’ disease had sodium levels <130 mmol/l as compared to only 8.2% of CAP patients with other etiologies (p < 0.001). In contrast, mean CT-ProVasopressin levels were not increased in patients with Legionnaires’ disease (39.4 pmol/l (±7) vs 51.2 pmol/l (±2.7), p = 0.43) (Figure 1).

**Figure 2:**
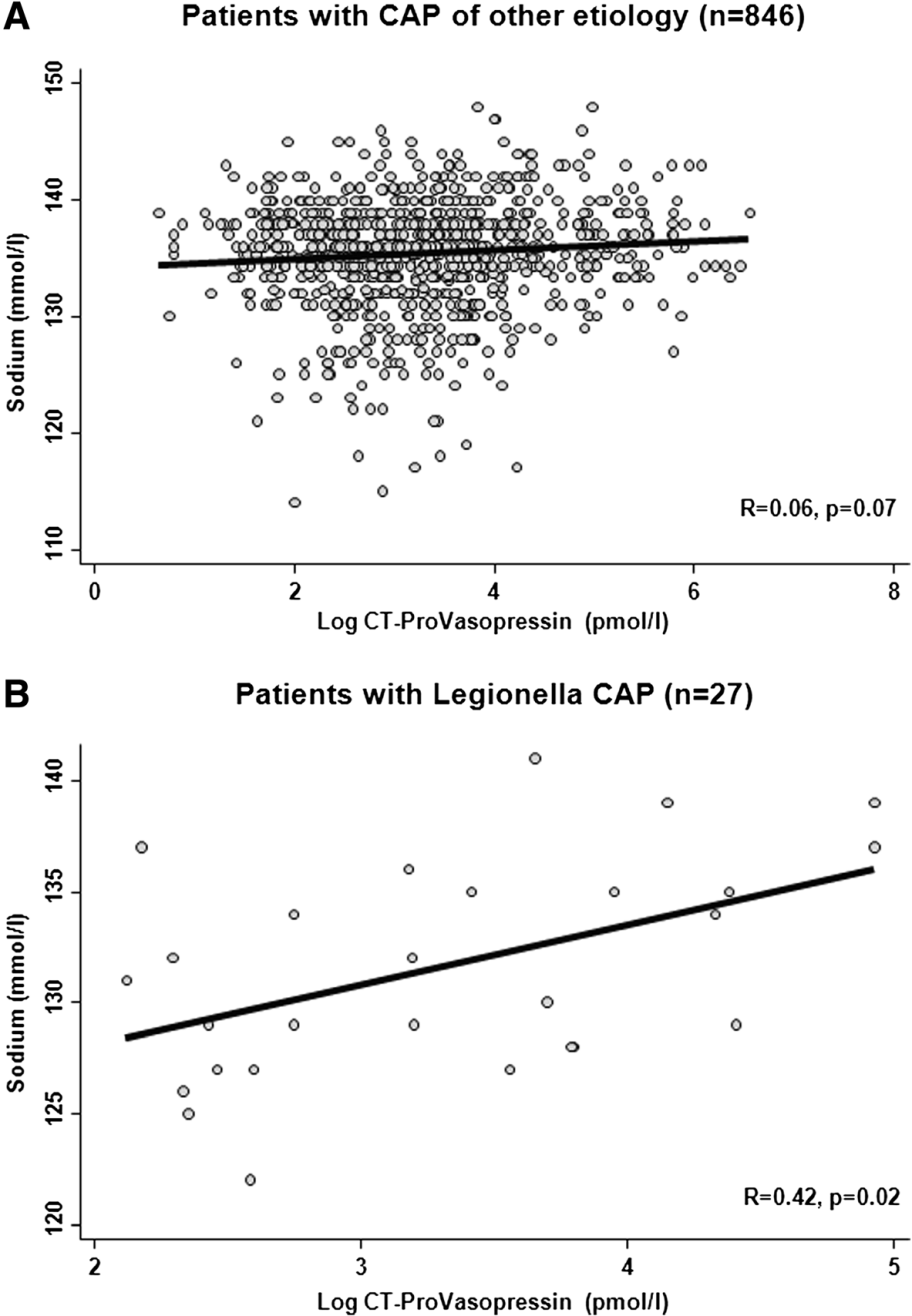
Correlation of sodium (A) and CT-ProVasopressin (B) levels in patients with CAP of other/unknown etiology and Legionnaires’ disease.

When comparing CT-ProVasopressin levels with sodium levels, we found no negative correlation in patients with Legionnaires’ disease which would be expected when ADH precursors were causatively related to hyponatremia. In contrast, ADH precursors showed a positive correlation with sodium levels (r = 0.42, p < 0.05) arguing against this hypothesis (Figure 2). Similarly, in patients with other CAP etiologies, there was a slight positive correlation of CT-ProVasopressin and sodium levels without reaching statistical significance (r = 0.06, p = 0.07).

To test the hypothesis that CT-ProVasopressin would be causal in the relationship of *Legionella* etiology and low sodium levels, we calculated multivariate regression models with stepwise inclusion of one or both parameters. There was again a strong association of *Legionella* etiology and low sodium levels (Table 2). This association was independent of age, gender, severity as assessed with the CURB65 score and markers of inflammation, infection and renal function. When also controlling for CT-ProVasopressin by adding this parameter into the regression model, the association of *Legionella sp.* etiology and low sodium was virtually unchanged, again suggesting that CT-ProVasopressin was not involved in the physiopathological pathway.

**Table 2:** Association of low sodium level (<130 mmol/L) and Legionnaires’ disease in univariate and multivariate logistic regression models

**Parameter**	**OR (95% CI)**	***p***
Univariate model	9.01 (95% CI 4.05, 20.01)	*<0.001*
*Legionella* (vs other etiology)		
Multivariate model*	7.74 (95% CI 3.26, 18.38)	*<0.001*
*Legionella* (vs other etiology)		
Multivariate model including CT-ProVasopressin†	7.53 (95% CI 3.17, 19.90)	*<0.001*
*Legionella* (vs other etiology)		

*adjusted for of age, gender, severity as assessed with the CURB65 score and markers of inflammation (CRP), infection (PCT) and renal function (creatinine).

**†**adjusted for above parameters as a well as CT-ProVasopressin.

### CT-ProVasopressin and severity of disease

**Figure 3:**
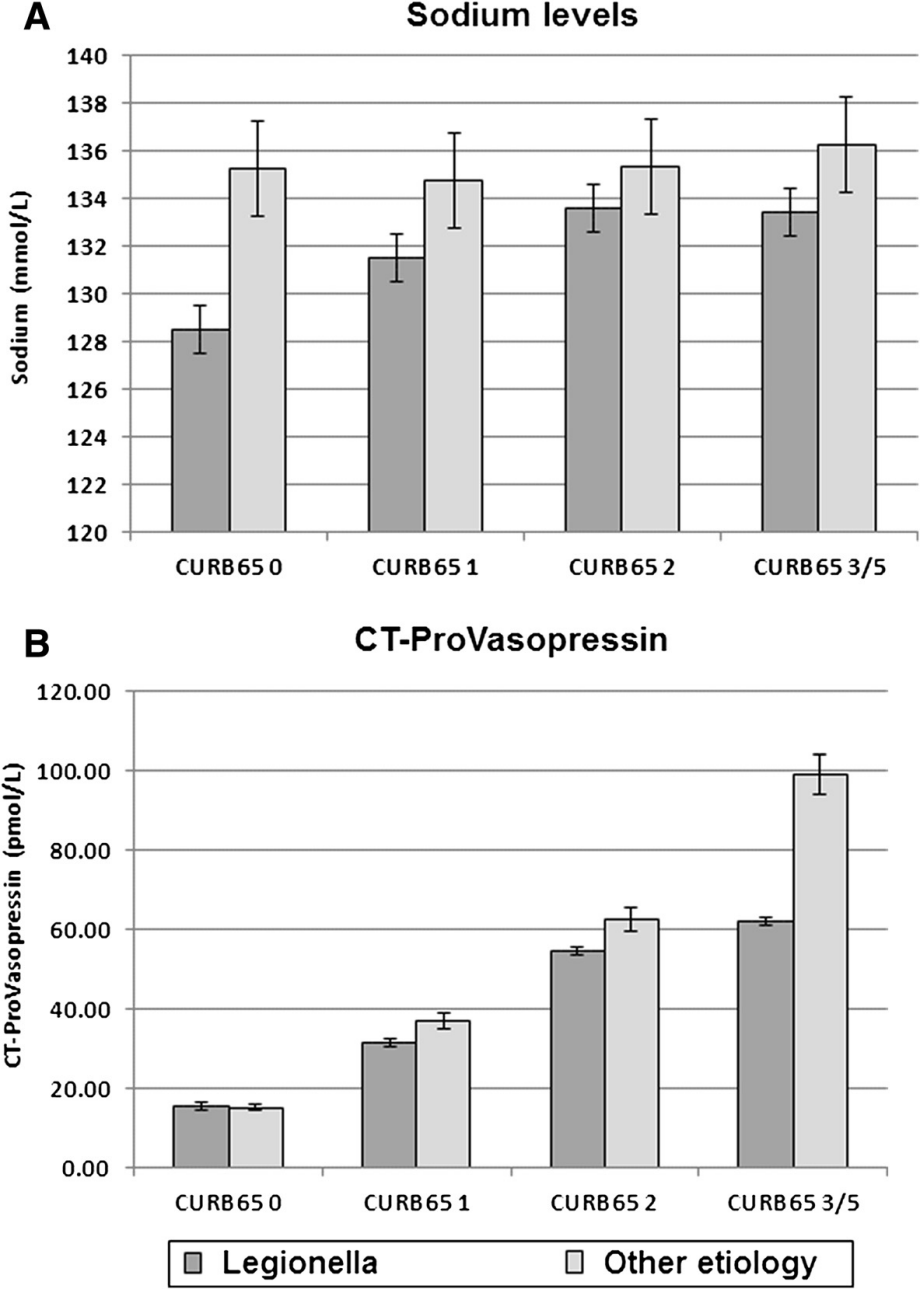
Plasma sodium (A) and CT-ProVasopressin (B) according to CAP severity assessed with the CURB65 score in patients with Legionnaires’ disease (dark grey) and patients with other CAP aetiology (light grey).

In patients with Legionnaires’ disease, and also in all other patients, sodium levels were not associated with severity of pneumonia assessed with the CURB65 score (*p = 0.14* in Legionnaires’ disease, *p = 0.3* in other CAP Figure 3A). However, patients showed a stepwise increase of CT-ProVasopressin with increasing CURB65 score (*p < 0.01* for both populations Figure 3B). A similar significant increase was found for PSI classes (r = 0.56, p < 0.05). Thus, CT-ProVasopressin was mainly increased in response to severity, and this was independent of blood sodium levels.

To investigate the prognostic potential of CT-ProVasopressin, we also investigated its association with mortality and ICU admission. In a multivariate logistic regression model including the CURB65 score, CT-ProVasopressin was an independent predictor for death (adjusted OR per decile increase in CT-ProVasopressin (pmol/L): 1.8 (95% CI 1.3-2.4), AUC 0.73) and for ICU admission (adjusted OR per decile increase in CT-ProVasopressin (pmol/L): 1.6 (95% CI 1.2-2.0), AUC 0.68). Thereby, CT-ProVasopressin significantly improved the CURB65 score for prediction of death (AUC improvement from 0.72 to 0.77, p < 0.05) and for ICU admission (AUC improvement from 0.64 to 0.69, p < 0.01).

## Discussion

Although textbooks commonly list Legionnaires’ disease as a differential diagnosis in patients with SIADH, scientific evidence linking low sodium levels commonly found in Legionnaires’ disease to SIADH is sparse [30,31]. The pathophysiologic differentiation is crucial, since SIADH should be treated with free water restriction, whereas sepsis requires volume replacement at the same time for hemodynamic management. Within this large study of consecutive patients with CAP of different etiologies and severities, we tested the hypothesis that low sodium levels in Legionnaires’ disease could be explained by inappropriately high levels of CT-ProVasopressin. Importantly, our results dismiss this hypothesis, since hyponatremic patients did not display high CT-ProVasopressin levels. In contrast there was a positive correlation between sodium and CT-ProVasopressin levels. This was also confirmed in a multivariate model where *Legionella sp.* etiology of CAP was a strong predictor for low sodium levels, independent of disease severity, which remained unchanged after inclusion of CT-ProVasopressin into the model again arguing against a causal pathway.

Within this secondary analysis of a former prospective study, we sought to explore the role of ADH in hyponatremia a finding more specific to Legionnaires’ disease compared to CAP of other etiologies. ADH is not routinely measured in the work-up of SIADH, partially because of the analytical challenges of ADH measurement. Instead, the diagnosis of SIADH is based on clinical features and includes hyponatremia and hypoosmolality in plasma while the patient is in an euvolemic state. Urine osmolality and sodium are inappropriately high in the absence of edema-forming states, thyroid and adrenal dysfunction [32]. Yet, with the recent availability of an immuno-assay measuring the more stable ADH precursor-peptide (CT-ProVasopressin) that is produced in equimolar ratio to ADH, this problem can now be addressed [33]. Clinical studies have shown a very high correlation of CT-ProVasopressin with ADH, demonstrating that CT-ProVasopressin indeed can be used a surrogate for ADH production in clinical practice [34-36]. Still, different distinct ADH patterns in patients with an established diagnosis of SIADH may exist which further challenge the interpretation of laboratory results [37]. First, a reset osmostat with a positive correlation of ADH and osmolality set for total ADH suppression lower than the physiologic osmolality threshold of about 280 mOsm/kg. This usually results in a constant and asymptomatic mild hyponatremia. Second, a constant and non-suppressible ADH secretion independent of plasma osmolality, caused by a constant ectopic or neurohypophyseal release. Third, hyponatremia with completely suppressed ADH secretion while still maintaining an inappropriate amount of antidiuresis and thus causing hyponatremia. In addition, in some patients no good correlation may be found between ADH levels and plasma osmolality, although patients formally fulfill the diagnosis of SIADH. As our study has focused on laboratory results only, these data need confirmation in a trial where patients volume status as well as urinary electrolytes are taken into account as well.

Few reports investigated ADH levels in CAP patients. A study including 28 adult CAP patients reported increased ADH levels and impairments in renal water excretion [6,38]. Similarly, in children with tuberculosis and respiratory syncytial virus (RSV) pneumonia, increased ADH levels were described and correlated with the degree of hypoxia and hypercapnia [5,39-44]. While, we also documented increased levels of CT-ProVasopressin in our study of CAP patients, there was no association of high ADH precursor levels and low sodium levels. Yet, CT-ProVasopressin showed a stepwise increase with increasing severity of disease. Thus, the subtle increase in ADH precursor level in CAP patients with hyponatremia is likely due to a stress response which overrules the osmoregulatory function. This finding is consistent with several observational studies demonstrating that high blood levels of CT-ProVasopressin are associated with adverse clinical course and mortality in patients with sepsis and respiratory infections [17,45,46]. The same correlation was shown for other hormones as Proadrenomedullin [47,48]. This may be explained by the fact that ADH production in the posterior pituitary gland is directly positively regulated through corticotropin-releasing hormone (CRH) and itself stimulates corticotropin secretion in the adrenal pituitary. ADH thus acts as a stress hormone, similar to cortisol, and retains fluids in the body during situations of acute stress. Based on our analysis, we believe that in severe disease the stress response *per se* is the dominant stimulus for the upregulation of ADH, and overrules the osmotic stimulation. Still, as evidenced by the positive correlation between sodium levels and ADH-precursors, the physiological osmoregulatory stimulus to ADH secretions seems to be intact in this patient population.

Other mechanisms may explain why patients with Legionnaires’ disease frequently have low sodium levels. First, studies have shown direct effects of cytokines on the kidney leading to renal salt loss [49-51]. Haines et al. postulated a direct nephrotoxic effect of *Legionella sp.*, in some cases leading to acute tubular necrosis or interstitial nephritis with salt loss [9]. Also, other natriuretic hormones may be important in this regard. Different studies have reported increased levels of atrial natriuretic peptide (ANP), as well as B-type-natriuretic peptide (BNP) in patients with pneumonia [52-54]. These hormones may contribute to low sodium levels via increased natriuresis.

The strengths of our study are the systematic assessment of sodium and CT-ProVasopressin in a large prospective study of patients with CAP of different etiologies. Yet, this study also has limitations. First, we did not measure mature ADH directly. However, clinical evidence suggests that CT-ProVasopressin is a good surrogate for ADH, and may thus be used instead in clinical routine [55]. Still, the ratio of CT-ProVasopressin to ADH plasma concentrations may be increased in patients with sepsis, suggesting that CT-ProVasopressin may somewhat overestimate ADH plasma concentrations in patients with severe medical conditions [18]. Second, we were not able to assess sodium and osmolarity measurements in urine limiting our model to the measurement of CT-ProVasopressin and correlating blood sodium levels. Our observations were rather on the population level and not on a patient level [16,37,56]. Last, there may have been unmeasured confounders in our study population such as the use of diuretics and aldosterone antagonist, which were not recorded.

## Conclusions

In conclusion, we confirm a high prevalence of hyponatremia in Legionnaires’ disease compared to other CAP etiologies. But our report does not lend support to the hypothesis of elevated ADH levels causing hyponatremia in Legionnaires’ disease nor in CAP of other etiology. Rather, ADH was upregulated in response to the stress of severe infection, which seemed to have overruled the osmoregulatory stimulus. Other ADH-independent mechanisms are more likely to cause salt loss during Legionnaires’ disease, such as direct renal effects of cytokines or toxins, as well as natriuretic hormones. As a consequence, free water restriction should not be pursued in this patient population, but rather replacement of salt losses by hydration and salt repletion.

## Abbreviations

ADH: Antidiuretic hormone; CAP: Community-acquired pneumonia; COPD: Chronic obstructive pulmonary disease; SIADH: Syndrome of inappropriate antidiuretic hormone secretion; PCT: Procalcitonin; CRP: C-reactive protein; WBC: White blood cell count.

## Competing interest

The initial trial was supported by grant SNF 3200BO-116177/1 and 32003B_135222 from the Swiss National Science Foundation. Dr. Schuetz was supported by a research grant from the Swiss Foundation for Grants in Biology and Medicine (Schweizerische Stiftung für medizinisch-biologische Stipendien, SSMBS, PASMP3-127684/1). Dr. Christ-Crain was supported by a grant of the Swiss National Science Foundation (PP00P3-12346).

No commercial sponsor had any involvement in design and conduct of this study, namely collection, management, analysis, and interpretation of the data; and preparation, decision to submit, review, or approval of the manuscript.

PS, MCC, WCA and BM received support from BRAHMS/Thermofisher and BioMerieux to attend meetings and fulfilled speaking engagements. BM has served as a consultant and received research support. All other authors declare that they have no competing interests.

## Authors’ contributions

MCC, BM, WZ and PS had the idea and initiated the study, SH, BM and PS performed the analyses and drafted the manuscript. All authors amended and commented on the manuscript and approved the final version. PS is the guarantor of the paper, taking responsibility for the integrity of the work as a whole, from incepton to published article.

## Pre-publication history

The pre-publication history for this paper can be accessed here:

http://www.biomedcentral.com/1471-2334/13/585/prepub
